# Practical considerations for the analysis of time-resolved x-ray data

**DOI:** 10.1063/4.0000196

**Published:** 2023-08-16

**Authors:** Marius Schmidt

**Affiliations:** Physics Department, University of Wisconsin–Milwaukee, Milwaukee, Wisconsin 53211, USA

## Abstract

The field of time-resolved macromolecular crystallography has been expanding rapidly after free electron lasers for hard x rays (XFELs) became available. Techniques to collect and process data from XFELs spread to synchrotron light sources. Although time-scales and data collection modalities can differ substantially between these types of light sources, the analysis of the resulting x-ray data proceeds essentially along the same pathway. At the base of a successful time-resolved experiment is a difference electron density (DED) map that contains chemically meaningful signal. If such a difference map cannot be obtained, the experiment has failed. Here, a practical approach is presented to calculate DED maps and use them to determine structural models.

## THE PHYSICAL BASIS OF DED MAPS, REAL SPACE REPRESENTATION

The need for a practical tutorial how to analyze time-resolved crystallographic (TRX) data led to a workshop at the 10th Annual international BioXFEL conference in San Juan, Puerto Rico in May 2023. The majority of the material used at this workshop is referenced here. This manuscript is intended as a tutorial for a practical approach to producing high-quality difference electron density maps from crystallographic data. In addition, it outlines how to use a difference electron density map to determine a molecular structure, which could be a structure of an intermediate or any other structure of interest. The tutorial is driven by the need to analyze small signal in difference maps caused by a weak extent of reaction initiation that is common to TRX experiments. In addition, it serves as a refresher and as an entry point to this fascinating field.

Related information is also found in the literature cited below. However, this manuscript does not explain how to process TRX data, neither Laue data (Moffat, 1989; Ren and Moffat, 1995) nor data collected by time-resolved serial crystallography (Aquila *et al.*, 2012; Tenboer *et al.*, 2014; and White, 2019). It also does not explain how to globally analyze the TRX data and extract chemical, kinetic mechanisms, and pure species structures from them. For this, the reader is referred to advanced literature (Schmidt *et al.*, 2003; Rajagopal *et al.*, 2004; Ihee *et al.*, 2005; Schmidt *et al.*, 2005; Schmidt, 2008; Jung *et al.*, 2013; Schmidt *et al.*, 2013; and Schmidt, 2019).

A description of a light-initiated reaction in a protein crystal is outlined below. The reaction proceeds through three intermediate species I_1_…,I_3_. At any time-point t during the reaction, the electron density ρ_t_ is a sum of the electron densities ρ_I1_…,ρ_I3_ of the pure intermediate species weighted by their respective fractional concentrations c_I1_…,c_I3_ plus the respective concentration c_ref_ of the reference state electron density ρ_ref_. The reference state denotes the one obtained without initiating the reaction, e.g., crystals were measured in the dark, or no substrate or ligand is added,

$$\rho_{t}=c_{I1,t}\rho_{I1}+c_{I2,t}\rho_{I2}+c_{I3,t}\rho_{I3}+c_{\text{ref},t}\rho_{\text{ref}}.$$ (1)

The fractional concentrations (or occupancies) are time-dependent. Their time-variations are called concentration profiles. The concentration profiles of the intermediates are dependent on the underlying chemical kinetic mechanism (Steinfeld *et al.*, 1985). The goal of any time-resolved experiment is to extract the physical properties of the pure species, such as spectra or structures in conjunction with the chemical, kinetic mechanism that gives rise to the concentration profile. From Eq. (1), one can see that there are 6 unknowns, namely, ρ_I1_ … ρ_I3_ and the c_I1_ … c_I3_. ρ_ref_ can be measured by a separate experiment without reaction initiation. C_ref_ follows from mass conservation as

$$c_{\text{total}}=1.0=c_{I1,t}+c_{I2,t}+c_{I3,t}+c_{\text{ref},t}$$ (2)

and

$$c_{\text{ref},t}=1.0-\left(c_{I1,t}+c_{I2,t}+c_{I3,t}\right).$$ (3)

POBy inserting Eq. (3) into (1), one obtains

$$\begin{array}{c} \rho_{t}=c_{I1,t}\rho_{I1}+c_{I2,t}\rho_{I2}+c_{I3,t}\rho_{I3}+\left(1.0-c_{I1,t}-c_{I2,t}-c_{I3,t}\right)\rho_{\text{ref}}=c_{I1,t}\underset{\Delta \rho_{I1}}{\underset{︸}{\left(\rho_{I1}-\rho_{\text{ref}}\right)}}+c_{I2,t}\underset{\Delta \rho_{I2}}{\underset{︸}{\left(\rho_{I2}-\rho_{\text{ref}}\right)}}+c_{I3,t}\underset{\Delta \rho_{I3}}{\underset{︸}{\left(\rho_{I3}-\rho_{\text{ref}}\right)}}+\rho_{\text{ref}} \end{array}.$$ (4)

The electron density at time t is the sum of the DED maps of the intermediate species weighted by their respective fractional concentrations plus the electron density of the reference state. Notably, ρ_ref_ is not multiplied with its respective fractional concentration because this has been eliminated by the law of mass conservation [Eqs. (2) and (3)].

Finally, by subtracting ρ_ref_, one obtains

$$\rho_{t}-\rho_{\text{ref}}=\underset{\text{DED}\;\text{map}}{\underset{︸}{\Delta \rho_{t}}}=c_{I1,t}\Delta \rho_{I1}+c_{I2,t}\Delta \rho_{I2}+c_{I3,t}\Delta \rho_{I3}.$$ (5)

The DED map Δρ_t_ is measurable (see next paragraph). It is a linear combination of time independent difference maps of the intermediates Δρ_I1_… Δρ_I3_ weighted by their respective fractional concentrations. There is no contribution of the reference state to the difference map. As in any time-resolved experiment, the time-information is exploited to (i) gain information about the time-dependent concentrations (the concentration profiles) of the intermediates and (ii) extract the electron densities of the time-independent species structures.

## THE PHYSICAL BASIS OF DED MAPS, RECIPROCAL SPACE REPRESENTATION

A DED map is generated with the help of difference structure factors (**DF**) that consist of difference structure factor amplitudes Δ|F| that are obtained by subtracting reference structure factor amplitudes $|F{|}_{\text{ref}}$ from $|F{|}_{t}$ measured at time t after reaction initiation. $\Delta {\left|F\right|}_{t}$ are combined with the phase derived from a good (well refined) model of the reference state

$$\mathrm{DF}=\Delta {\left|F\right|}_{t}e^{i\varphi_{r\text{ef}}}.$$ (6)

Note, in the following, a structure factor is shown in bold-face. The structure factor amplitude is shown in light-face. Here, the difference amplitude can be positive or negative. If there is an objection that an amplitude must be positive, a negative amplitude can easily be set to positive by adding 180° (π) to the phase.

**FIG. 1.**
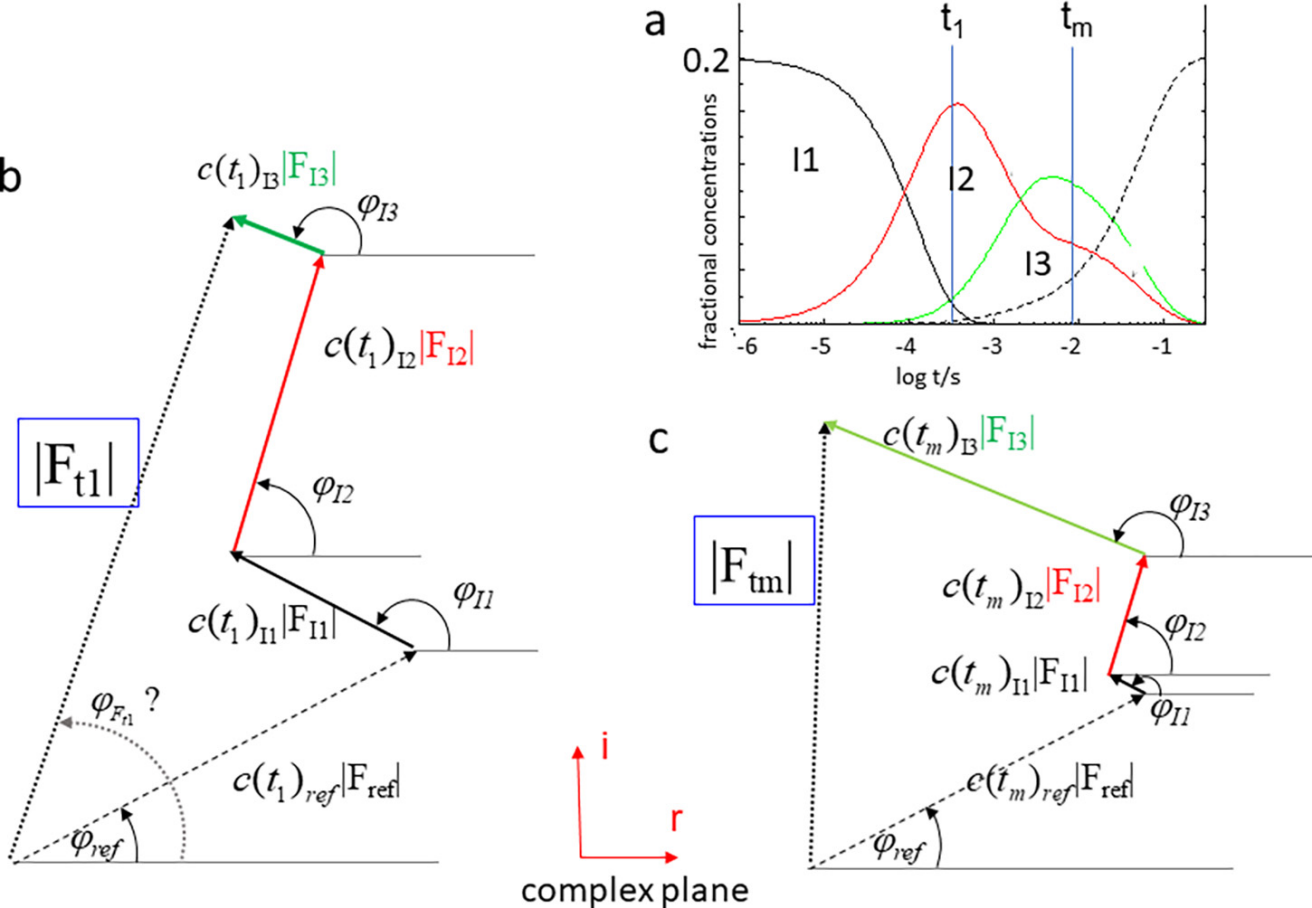
Time-dependent structure factors. The structure factor **F_t_** with ampitude |F_t_| and an unknown phase (*φ_Ft_*) is considered for two time points t_1_ and t_m_. Representation in the complex plane (Argand diagram). (a) Concentration profile of three intermediates. Fractional concentrations are plotted against log t. (b) Situation at time point t_1_. **F_t_** is obtained by a summation of structure factors of the reference state (**F**_ref_) plus those of the intermediate species (**F**_I1_ … **F**_I3_**).** All structure factors are weighted by their respective fractional concentrations (c_ref_, c_I1_ … c_I3_). (c) Situation at time point t_m_. The structure factor amplitude |F_tm_| is different in length from that in (b). Phases except that of the reference state are unknown. The structure factor amplitudes of the intermediate species are also unknown. All fractional concentrations are equally unknown. The structure factor of the reference state (**F_ref_**) is known with amplitude and phase, and the fractional concentration of the reference state can be deduced using the mass conservation law [Eq. (2)]. Only the amplitude |F_t_| of the structure factor **F**_t_ is measurable for each time point. The phase is not known [denoted by the question mark in (b)].

In general, a time-resolved crystallographic experiment consists of two steps. (i) Reference structure factor amplitudes $|F{|}_{\text{ref}}$ are collected from protein crystals where a reaction has not been started. This can be done for example by exposing the crystals in the dark ahead of a pump-probe experiment. In step (ii), time-dependent structure factor amplitudes $\left|F_{t}\right|$ are collected at a time delay t after a reaction is initiated, for example, by an intense laser light pulse or by mixing with the substrate. $\left|F_{t}\right|$ is the amplitude of the time-dependent structure factor $F_{t}$. $F_{t}$ is the reciprocal space equivalent of Eq. (1). Figure 1 shows that how $F_{t}$ is constructed from the structure factors of the intermediates and the reference state. After processing, two crystallographic datasets are obtained, one for the reference state, and another that probes the progress of the reaction at time-point t. (More datasets can be obtained at any other time-point.) Both datasets consist of a long list of Miller indices, structure factor amplitudes, and their measurement errors. Data are typically stored in a binary format called the mtz-format (after the progenitors McLaughlin, Terry, and Zelinka, see also the IUCr Commission on Crystallographic Computing) that is standard to the collaborative project number 4 (CCP4) suite of programs (Winn *et al.*, 2011).

**FIG. 2.**
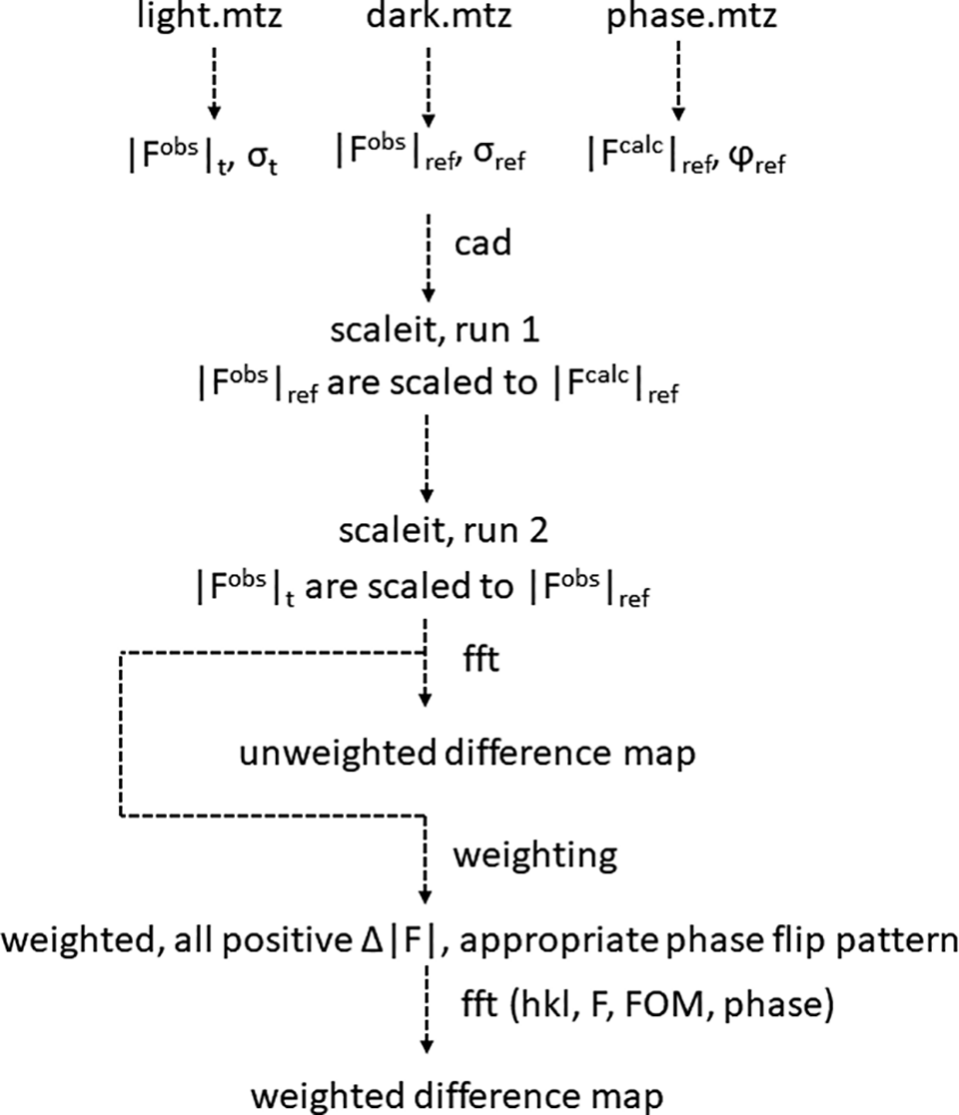
From structure factor amplitudes to weighted DED maps. Here, datasets from a pump-probe experiment are evaluated. Observed structure factor amplitudes and their experimental errors (sigmas) are obtained from crystals that were excited by laser light (light.mtz). A reference dataset is collected in the dark (dark.mtz). Phases and calculated amplitudes are available after refinement. Scaleit from the ccp4 suite of programs is used to scale the data together. After scaling, unweighted DED maps can be calculated. Weighted, positive difference amplitudes as well as an appropriate phase flip pattern will be provided to calculate weighted difference maps. The input to “fft” is equivalent to that required for the calculation of a conventional “figure of merit (FOM)” weighted electron density map.

Figure 2 shows a flow chart how to calculate a difference map from measured data. The mtz-file that contains the reference structure factor amplitudes $\left|F_{\text{ref}}^{\text{obs}}\right|$ is called dark.mtz that with the time-resolved data $\left|F_{t}^{\text{obs}}\right|$ is called light.mtz. This mimics the result of a pump-probe TRX experiment where the reaction is started in the crystals with laser light pulses. The mtz files could easily be called water.mtz and ligand.mtz where both files might have originated from a substrate diffusion experiment (Schmidt, 2013; Olmos *et al.*, 2018), or given any other name. A third mtz file is required that contains the phases of the reference state, called here φ_ref_. This mtz file is obtained by refining a reference state model against the structure factor amplitudes contained in the dark.mtz file using standard refinement programs, such as refmac (Murshudov *et al.*, 2011) or phenix (Adams *et al.*, 2010; Liebschner *et al.*, 2019). The reference model should be as complete as possible including all water molecules and other ions or ligands. The refinement should provide a set of the best reference phases possible. It also provides a set of calculated structure factor amplitudes $\left|F_{\text{ref}}^{\text{calc}}\right|$ that fit the observed structure factor amplitudes of the reference state $\left|F_{\text{ref}}^{\text{obs}}\right|$ as accurately as possible. The $\left|F_{\text{ref}}^{\text{calc}}\right|$ are by definition on the absolute scale.

Difference map calculations rely on proper scaling of the $\left|F_{\text{ref}}^{\text{obs}}\right|$ to the $\left|F_{t}^{\text{obs}}\right|$. In general, this is done by applying a resolution dependent scaling model that can consist of scale factors and isotropic or anisotropic B-factors (Evans, 2006; Dalton *et al.*, 2022). Typically, the quality of the $\left|F_{\text{ref}}^{\text{obs}}\right|$ dataset is better than that of the $\left|F_{t}^{\text{obs}}\right|$ data. This is because the reaction in the crystals produces disorder that is larger than that in the crystals at rest. The time-dependent intensities (or amplitudes) are corrected during scaling. Wilson plots with the ${\left|F_{t}^{\text{obs}}\right|}^{2}$ and the ${\left|F_{\text{ref}}^{\text{obs}}\right|}^{2}$ have approximately the same slope after scaling. Disorder of individual atoms engaged in the reaction causes the magnitudes of the positive difference features to be often smaller than those of the negative ones.

To perform the scaling on the absolute scale, the $\left|F_{\text{ref}}^{\text{obs}}\right|$ are scaled to the $\left|F_{\text{ref}}^{\text{calc}}\right|$ in a first step. This is equivalent of determining a crystallographic R-factor [R_cryst_, Eq. (7)] during refinement. Once the $\left|F_{\text{ref}}^{\text{obs}}\right|$ are on the absolute scale, the $\left|F_{t}^{\text{obs}}\right|$ are scaled to them. The progress of the scaling can be monitored by an R_scale_ [Eq. (7)] that is calculated in a similar way as the familiar R_cryst_

$$R_{\text{cryst}}=\frac{\sum_{\text{hkl}}\left|\left|F^{\text{obs}}\right|-\left|F^{\text{calc}}\right|\right|}{\sum_{\text{hkl}}\left|F^{\text{obs}}\right|};\;R_{\text{scale}}=\frac{\sum_{\text{hkl}}\left|\left|F_{t}^{\text{obs}}\right|-\left|F_{\text{ref}}^{\text{obs}}\right|\right|}{\sum_{\text{hkl}}\left|F_{t}^{\text{obs}}\right|}.$$ (7)

Once the data are properly scaled, the $\left|F_{\text{ref}}^{\text{obs}}\right|$ are subtracted from the $\left|F_{t}^{\text{obs}}\right|$ to obtain a dataset of $\Delta {\left|F\right|}_{t,\text{obs}}$. It is worthwhile to note that crystallographic R-factor (R_cryst_) is typically on the order of 15%–20% caused by inaccuracies in the structural model. Therefore, the scaling of the observed to the calculated data results in a similar unfavorable R_scale_-factor. It is shown further down that the quality of the difference maps decisively depends on whether the sign (positive or negative) of the differences can be accurately determined. When the R_scale_ is large, this sign cannot be accurately determined. That makes the discovery of small signal difficult even when the so-called mF^obs^–DF^calc^ difference maps are used (m is the figure of merit and D is a weighting factor that accounts for model errors) (Read, 1986). In contrast, scaling two observed datasets results in much lower R_scale_-factors on the order of 5%–10%. This leads to much more accurate Δ|F|(and their signs) that are meaningful even when the extent of the reaction initiation is low (e.g., smaller than 10%).

Monitoring the R_scale_ is important to predict the success of a time-resolved experiment. When the data quality is poor because (i) not enough diffraction patterns are collected, (ii) unsuitable data processing parameters are employed, (iii) the detector geometry has not been determined correctly, or (iv) the detector itself is not in good working condition, the R_scale_ will be elevated caused by both systematic and experimental error in the collected data. Then, a meaningful difference electron density map cannot be obtained. Improvements in data processing or collecting more diffraction patterns (whatever is relevant for the particular experiment) may be necessary. From experience, R_scale_ factors near of smaller than 10% are found to be necessary to result in good difference maps with meaningful signal.

The $\Delta {\left|F\right|}_{t}$ are then combined with the reference state phases φ_ref_ to difference structure factors [**DF**, Eq. (6)], from which a difference map can be calculated by Fourier summation [Eq. (8)]. The difference electron density is represented on only half the absolute

$$\Delta \rho_{t}(\vec{R})=\frac{1}{V}\sum_{\vec{h}=\text{hkl}}\overset{\mathrm{DF}}{\overset{︷}{\Delta {\left|F\right|}_{t,\text{obs}}\;{\text{exp}}^{i\varphi_{\text{ref}}}}}\;{\text{exp}}^{2\pi i\vec{h}\cdot \vec{R}}.$$ (8)

$\vec{R}$ denotes the position in the unit cell (in fractional coordinates), and $\vec{h}$ are the Miller indices or integer multiples of them. Equation (8) is the relevant, governing equation, which provides the experimental result of a time-resolved crystallographic experiment. From a crystallographic point of view, Eq. (8) is incorrect and cannot be understood at this point. Apart from the claim that the difference electron density is represented on half the absolute scale, the biggest concern is the utilization of the reference phase in Eq. (8) instead of the phase of the (true) difference structure factor. An additional concern is that the amplitudes Δ|F| have been calculated by subtracting amplitudes rather than structure factors. As such the **DF** appears to be inapt for the calculation of a difference map.

## THE DIFFERENCE FOURIER APPROXIMATION

**FIG. 3.**
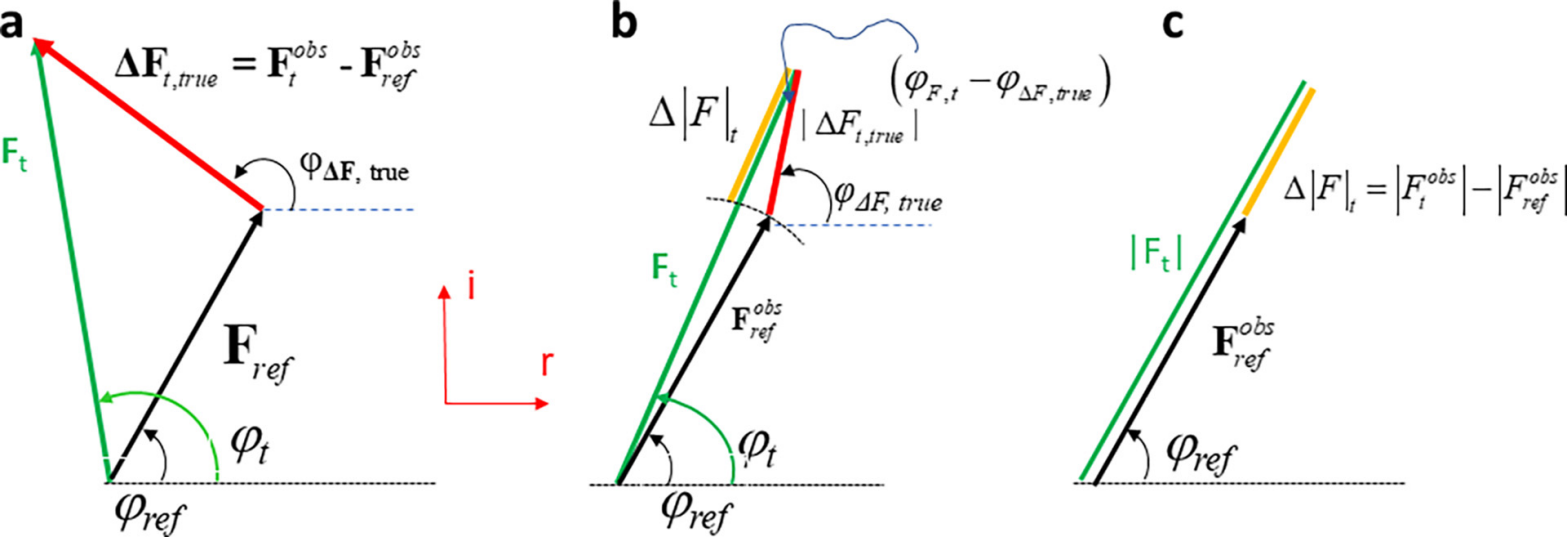
Argand diagrams to determine difference structure factors. (a) The true difference structure factor **ΔF_true_** (red arrow) with amplitude and phase φ_true_ is determined by vector subtraction of the structure factors **F_t_** and **F_ref_**. Although **F**_ref_ can be determined (by measurement and phases from a refined model), the phase φ_t_ remains unknown. The true difference structure factor cannot be calculated. (b) The difference approximation: since structural changes are small the difference between φ_t_ and φ_ref_ is small. The orange structure factor amplitude Δ|F|_t_ is obtained by projecting the true difference structure factor onto **F**_t_. (c) The length of the projection (orange) can be measured (estimated) by subtracting $\left|F_{\text{ref}}\right|$ from $\left|F_{t}\right|$. The difference structure factor **DF** now consists of the amplitude Δ|F|_t_ (which can be positive or negative) and the phase of the reference state as in Eq. (6).

Figure 1 shows that the phase of the structure factor **F_t_** is very difficult if not impossible to be measured during the time the reaction proceeds in the protein crystal. For the calculation of a true DED map, true difference structure factors **ΔF_true_** = **F**_t_ – **F**_ref_ are required [Fig. 3(a)]. **ΔF_true_** is obtained by subtracting the structure factor of the reference state from the time-dependent structure factor as a vector in the complex plane [Fig. 3(a)]. Then, the true DED map is calculated as

$$\Delta \rho_{\text{true}}(\vec{R})=\frac{1}{V}\sum_{\vec{h}=\text{hkl}}|\Delta F_{\text{true}}|\;{\text{exp}}^{i\varphi_{\Delta F,t\text{rue}}}\;{\text{exp}}^{2\pi i\vec{h}\cdot \vec{R}}.$$ (9)

Note, this time, $\Delta \rho_{\text{true}}$ is on the absolute scale when true difference structure factors **ΔF**_true_ are used with amplitude |ΔF_true_| and phase φ_ΔF,true_ [Fig. 3(a)]. Here, attention to detail is required: |ΔF_true_| (the vertical bars enclose the entire ΔF_true_) is the amplitude of the true difference structure factor generated by subtraction of two vectors in the complex plane [Fig. 3(a)]. However, as mentioned (Fig. 1), the phase φ_ΔF,true_ cannot be determined, and the difference structure factor **ΔF**_true_ cannot be calculated. This would be the end of any attempt to determine a difference map, if there would not be an approximation that makes it possible that instead of the true difference structure factor an observed difference structure factor amplitude could be used together with the reference (dark) state phase.

The difference approximation holds [Fig. 3(b)] when structural changes are small. It is not really clear what small means, but it is certainly fulfilled if only a few atoms of a 1000 atom molecule change their positions. Then, the phase difference between the dark and time-dependent structure factors is also small. When the true difference structure factor amplitude is projected onto **F**_t_ [Fig. 3(b), red and orange bars], the projection can be evaluated by using the cosine

$$\Delta {\left|F\right|}_{t}\approx \left|\Delta F_{\text{true}}\right|\;\text{cos}\;\left(\varphi_{F,t}-\varphi_{\Delta F,\text{true}}\right).$$ (10)

Equation (10) is the decisive equation. The $\Delta {\left|F\right|}_{t}$, which can be measured [Fig. 3(c)], is related to $\left|\Delta F_{\text{true}}\right|$ through this equation. The remaining calculation follows the derivation outlined in Drenth (1999). In the Drenth, $\Delta {\left|F\right|}_{t}$ is denoted $\Delta {\left|F\right|}_{\text{iso}}$, $\left|\Delta F_{\text{true}}\right|$ is denoted $\left|F_{H}\right|$, and $\text{cos}\;\left(\varphi_{F,t}-\varphi_{\Delta F,\text{true}}\right)$ is denoted $\text{cos}\;\left(\alpha_{PH}-\alpha_{H}\right)$. The factor ½ arises when the cosine is expressed as a sum of complex numbers (exponentials) as $\text{cos}\;\alpha =\frac{1}{2}\left(e^{i\alpha }+e^{-i\alpha }\right)$. Equation (6) then becomes

$$\Delta {\left|F\right|}_{t}e^{i\varphi_{r\text{ef}}}\approx \frac{1}{2}\left|\Delta F_{\text{true}}\right|\left[e^{i\left(\varphi_{F,t}-\varphi_{\Delta F,t\text{rue}}\right)}\times e^{i\varphi_{\text{ref}}}+e^{-i\left(\varphi_{F,t}-\varphi_{\Delta F,\text{true}}\right)}\times e^{i\varphi_{\text{ref}}}\right].$$ (11)

${\left|\Delta F\right|}_{\text{true}}$ is combined with its phase factor $e^{i\varphi_{\Delta F,\text{true}}}$ to the difference structure factor $\Delta F_{t\text{rue}}$, and with the conjugated phase factor $e^{-i\varphi_{\Delta F,\text{true}}}$ to $\Delta F_{\text{true}}^{*}$

$$\Delta {\left|F\right|}_{t}e^{i\varphi_{\text{ref}}}\approx \frac{1}{2}\Delta F_{\text{true}}\left(e^{-i\varphi_{F,t}}\times e^{i\varphi_{\text{ref}}}\right)+\frac{1}{2}\Delta F_{\text{true}}^{*}\left(e^{i\varphi_{F,t}}\times e^{i\varphi_{\text{ref}}}\right).$$ (12)

By acknowledging that the phase φ_t_ and φ_ref_ are essentially equal [hence the phase difference is small, Fig. 3(b)], an equation is derived that relates the measured (observed) difference structure factor **DF** (with amplitudes $\Delta {\left|F\right|}_{t}$ and phases φ_ref_) to the true difference structure factor

$$\overset{\mathrm{DF}}{\overset{︷}{\Delta {\left|F\right|}_{t,\text{obs}}\;{\text{exp}}^{i\varphi_{\text{ref}}}}}=\frac{1}{2}\Delta F_{\text{true}}+\underset{\text{noise}}{\underset{︸}{\frac{1}{2}\Delta F_{\text{true}}^{*}\;{\text{exp}}^{2i\varphi_{\text{ref}}}}}.$$ (13)

With the notion that the phase of the difference structure factor **ΔF**_true_ is not correlated with the reference phase, the second term on the right-hand side of Eq. (13) averages out in a Fourier summation. Equation (13) is the mathematical counterpart of the difference Fourier approximation. Equation (8) can be derived this way from first principles. The reason why Δρ is only represented on half the absolute scale and the justification that the reference phase can be used is now understood, because the difference structure factor **DF** with measured amplitudes Δ|F| and model phases φ_ref_ is approximately $\frac{1}{2}\Delta F_{\text{true}}$. Accordingly, a difference map calculated with the observed differences and the phases of the reference state is a true difference map with DED features on ½ the absolute scale and some additional noise caused by the second term on the right-hand side of Eq. (13). Since $\Delta {\left|F\right|}_{t}$ can be accurately measured [Fig. 3(c)], the experimental DED map is very sensitive to structural and occupancy changes (Henderson and Moffat, 1971).

## NECESSITY TO WEIGHT DIFFERENCE STRUCTURE FACTOR AMPLITUDES

In early times of time-resolved crystallography, DED maps were determined from quite noisy data. Substantial effort has been made to identify sources of noise and systematics that might influence the quality of the DED maps. In a ground laying paper, Ursby and Bourgeois (1997) determined a weighting scheme to extract the best difference map given poorly measured data and errors in the structural model required to calculate φ_ref_. The two main errors identified were difference structure factor amplitudes determined (i) from large amplitudes as well as (ii) from poorly measured amplitudes with large experimental error (sigma) values. Condition (i) arises, since large amplitudes also carry, on an absolute scale, large error values. If two large numbers are subtracted, large false positive and negative differences can likely arise. Large intensities (and amplitudes) are usually measured at low resolution. Low-resolution difference structure factor amplitudes generate a rolling DED landscape with high crests and deep valleys in which the high-resolution difference features are located. The contour level is determined by fluctuations in the unit cell that determine the sigma value of the DED map. True DED features tend to be obscured by the valleys or crests of the low-resolution DED landscape. Poorly measured amplitudes (ii) can also result in false positive or negative differences but affect mostly high-resolution differences. In both cases, false positive or negative differences may occur, that deteriorate features in the DED map. It is, therefore, desirable to weigh down either large differences or those with large experimental errors. Based on the statistical considerations by Ursby and Bourgeois (Ursby and Bourgeois, 1997), Zhong Ren and colleagues developed a largely simplified, practical weighting factor that can be used to correct for both conditions (i) and (ii) (Ren *et al.*, 2001). The weighting factor is calculated for each difference structure factor amplitude with index hkl,

$$w_{\text{hkl}}=\frac{1}{1+\frac{{\left(\Delta {\left|F\right|}_{\text{hkl}}\right)}^{2}}{\left\langle {\left(\Delta \left|F\right|\right)}^{2}\right\rangle }+\frac{{\left(\sigma_{\Delta {\left|F\right|}_{\text{hkl}}}\right)}^{2}}{\left\langle {\left(\sigma_{\Delta \left|F\right|}\right)}^{2}\right\rangle }}.$$ (14)

The denominator consists of three terms. The second term considers large difference amplitudes. It down-weights them, if they are much larger than the average difference $\left\langle {\left(\Delta \left|F\right|\right)}^{2}\right\rangle$ found in the dataset. The third term deals with sigmas. Again, here the weighting factor becomes small when the sigma of the difference structure factor amplitude σ_Δ|F|_ is much larger than the average sigma $\left\langle {\left(\sigma_{\Delta \left|F\right|}\right)}^{2}\right\rangle$ found in the dataset of difference structure factor amplitudes. (Note that the σ_Δ|F|_ is determined by error propagation from the individual measurement errors of the amplitudes used to calculate the Δ|F|.) This weighting scheme can be easily implemented in a computer program and does not need additional information, such as coordinate errors. In the original article by Ren *et al.*, the values in terms 2 and 3 were not squared. The author of this article uses the squared values in publications he authored and coauthored and obtained good results. Other weighting schemes are discussed by De Zitter *et al.* (De Zitter *et al.*, 2022).

The difference structure factor amplitudes are now weighted as

$$\Delta {\left|F\right|}_{\text{hkl},w}=\frac{w_{\text{hkl}}\cdot \Delta {\left|F\right|}_{\text{hkl}}}{\left\langle w\right\rangle }.$$ (15)

They are divided by the average weight to maintain the absolute scale established after scaling. Weighted DED maps are calculated accordingly

$$\Delta \rho_{w}=\frac{1}{V}\sum_{\vec{h}=\text{hkl}}\Delta {\left|F\right|}_{w}\;{\text{exp}}^{i\varphi_{\text{ref}}}\;{\text{exp}}^{2\pi i\vec{h}\cdot \vec{R}}.$$ (16)

**FIG. 4.**
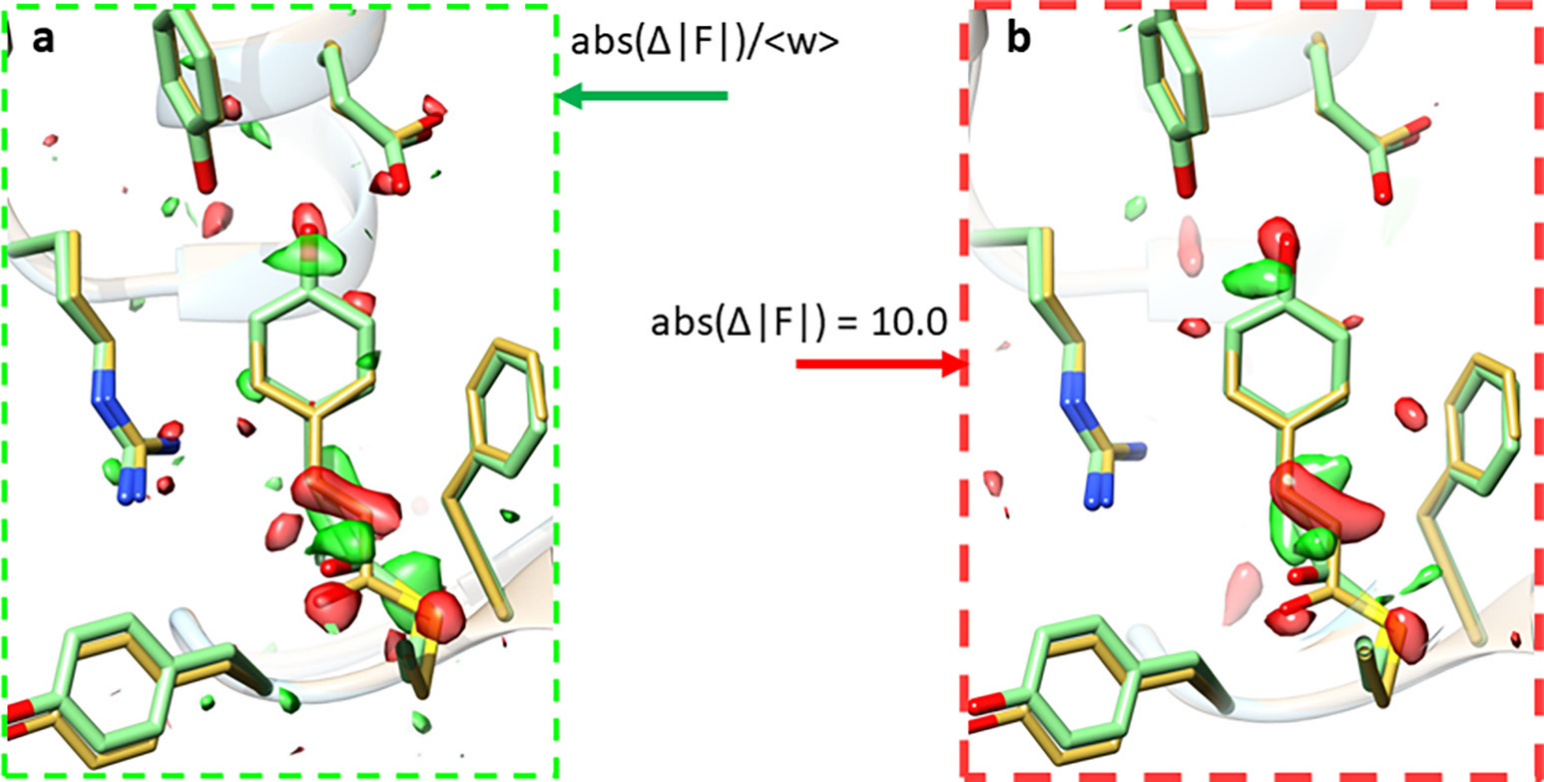
DED maps. (a) Calculated with weighted (but positive) difference structure factor amplitudes and the correct phase flip pattern [Eq. (17)]. (b) The F10 difference map is calculated with all difference structure factor amplitudes set to 10.0 and all weights set to 1.0. The correct phase flip pattern from (a) is maintained. The resulting DED map is very similar to that in (a).

An exemplary weighted time-dependent DED map is shown in Fig. 4(a). The data for this map were collected at the CXI instrument (Liang *et al.*, 2015) at the Linac coherent light source (LCLS) during a pump-probe experiment on photoactive yellow protein (Pande *et al.*, 2016).

## DED MAP FUN

Difference structure factors are required to calculate DED maps. They consist of measured positive and negative difference amplitudes and phases φ_ref_ of the dark state [see Eq. (13)]. However, as mentioned, a convenient feature in crystallography is that negative amplitudes can be always converted to positive ones by flipping the phase by 180°,

$$-\Delta \left|F\right|e^{i\varphi_{\text{ref}}}=+\Delta \left|F\right|e^{i\left(\varphi_{\text{ref}}+\pi \right)}.$$ (17)

Therefore, a difference map can be calculated by using exclusively positive difference structure factor amplitudes and a pattern of reference phases or reference phases flipped by 180°. For good book-keeping practice, all difference structure factor amplitudes can be made positive on the cost of flipping the reference phase once required. Maintaining negative difference structure factor amplitudes should be discouraged. Then, a DED map is calculated exactly in the same way as a conventional electron density map. This gives some room for experimentation. Suppose that the phase flip pattern has been determined by setting all negative difference structure factor amplitudes to positive and flip the phase when necessary. One could then imagine replacing all amplitudes with a fixed, positive value (e.g., 10) and all the weights with unity (1.0). A simple unix command would do this: awk '{print $1,$2,$3,” 10.0 1.0 “, $6}' difference.phs > differenceF10.phs. Here, hkl is assumed to occupy column 1, 2, and 3, and the phases (either the reference phase or the one flipped by 180°) are in column 6 of the hkl file.

From the new file differenceF10.phs, a DED map (F10) can be calculated and compared to the observed difference map determined from the appropriate amplitudes and weights. Surprisingly, the two difference maps are almost identical [compare Figs. 4(a) and 4(b)]. If one would observe the F10 difference map during a LCLS beamtime, one would already be satisfied, since the experiment worked. This clearly outlines that the determination of a correct phase-flip pattern (either dark phase or the phase flipped by 180°) is sufficient to produce a good difference map. The art is to determine the phase-flip pattern correctly in the presence of experimental noise. If this pattern deteriorates caused by poorly measured structure factor amplitudes, the signal in the DED map deteriorates accordingly (Schmidt *et al.*, 2003). This is the reason that weighting is important (see above). The goal is to down-weigh the contribution by potentially false phase flips to keep the difference signal as strong as possible.

## STRUCTURES FROM DED MAPS

DED maps are notoriously difficult to interpret. When atoms move away, negative DED features appear at the position of the reference state atomic positions. However, the void can be filled with atoms from an intermediate. Then, the DED map is flat at this position, although large structural changes have occurred. It would be advantageous if a conventional electron density map would be provided for structure interpretation and refinement. Such an electron density map can be obtained by extrapolation (Genick *et al.*, 1997). The physical basis of extrapolation can be easily understood from real space considerations. Consider the presence of only one intermediate. Then, the observed DED map can be written as the DED map of the intermediate weighted by its corresponding occupancy c_I1_ [analogous to Eq. (4)],

$$\Delta \rho_{t}=c_{I1,t}\Delta \rho_{I1}.$$ (18)

By expanding the left and right hand side with the definition

$$\left(\rho_{t}-\rho_{\text{ref}}\right)=c_{I1,t}\left(\rho_{I1}-\rho_{\text{ref}}\right)$$ (19)

and solving for ρ_I1_, one obtains

$$\rho_{I1}=\rho_{\text{ref}}+\frac{1}{c_{I1,t}}\left(\rho_{t}-\rho_{\text{ref}}\right)$$ (20)

or

$$\rho_{I1}=\rho_{\text{ref}}+\frac{1}{c_{I1,t}}\Delta \rho .$$ (21)

It should be mentioned at this point: Δρ is only determined on half the absolute scale using the measured amplitudes and the reference (dark) phases [see above, Eqs. (8) and (13)]. Therefore, for this equation to work with measured DED maps, twice the measured Δρ must be added for the extrapolation to remain related to 1/c_I1_. This needs to be kept in mind.

From Eq. (21) which is defined in real space, a corresponding reciprocal space equation can be derived

$$\left|F^{\text{ext}}\right|=\left|F_{\text{ref}}^{\text{obs}}\right|+N\left(\Delta {\left|F\right|}_{t}\right)$$ (22)

with N = 2/occupancy. $\left|F^{\text{ext}}\right|$ can become negative when $\left|F_{\text{ref}}^{\text{obs}}\right|$ is small, N is large, and $\Delta {\left|F\right|}_{t}$ is strongly negative. As mentioned, the factor 2 corrects for half the absolute scale on which the observed DED map is determined, meaning that with small occupancy values typically observed in a TRX experiment (e.g., 10%), the correct factor N becomes large (N = 20 for this example). Since the difference amplitudes may carry substantial experimental noise, a large N amplifies this noise. As a result extrapolated maps are noisy. However, already 5% occupancy might produce a DED map with significant DED features. The N_C_ to determine extrapolated structure factor amplitudes would be 40. The hesitation to accept large N factors because only small Ns and correspondingly large occupancies result in an acceptable extrapolated map may lead to gross errors in the interpretation of structural changes. A suggestion how to obtain a better extrapolated map is outlined further down.

**FIG. 5.**
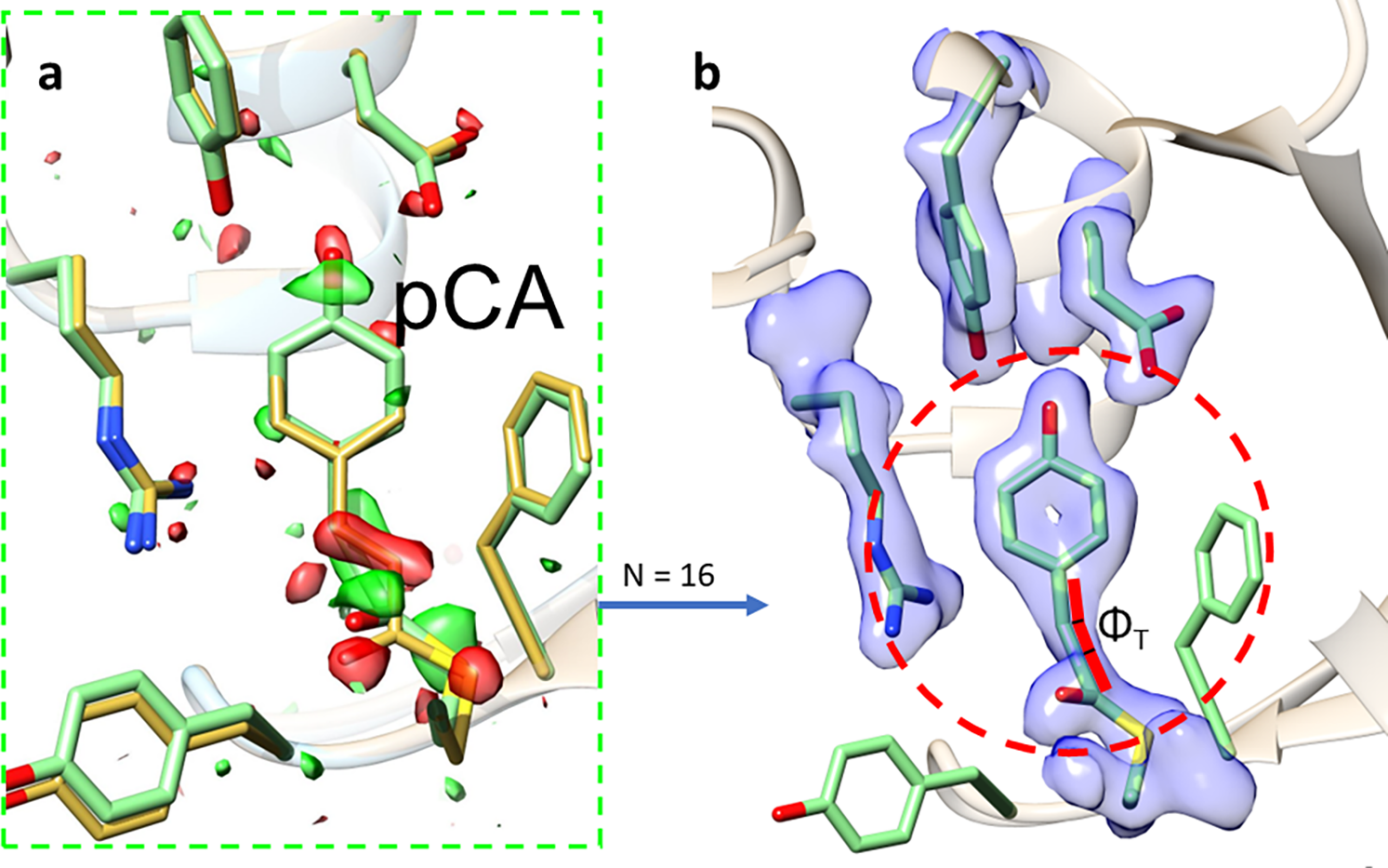
Extrapolated electron density from a DED map. (a) A DED map of PYP determined at 3 ps after reaction initiation. The yellow structure is the reference structure, and the green structure is determined from the extrapolated map. It fits the DED features perfectly. (b) Extrapolated map with N_C_ = 16. Data collected at LCLS. The region of interest used to integrate the negative electron density in the extrapolated map. The torsional angle Φ_T_ is marked in red. It is about 35°.

On a first glimpse, an extrapolated electron density map [such as the one shown in Fig. 5(b)] is calculated like a conventional map with the extrapolated amplitudes and the dark state phases

$$\rho^{\text{ext}}(\vec{R})=\frac{1}{V}\sum_{\vec{h}=\text{hkl}}\left|F^{\text{ext}}\right|\;{\text{exp}}^{i\varphi_{\text{ref}}}\;{\text{exp}}^{2\pi i\vec{h}\cdot \vec{R}}.$$ (23)

**FIG. 6.**
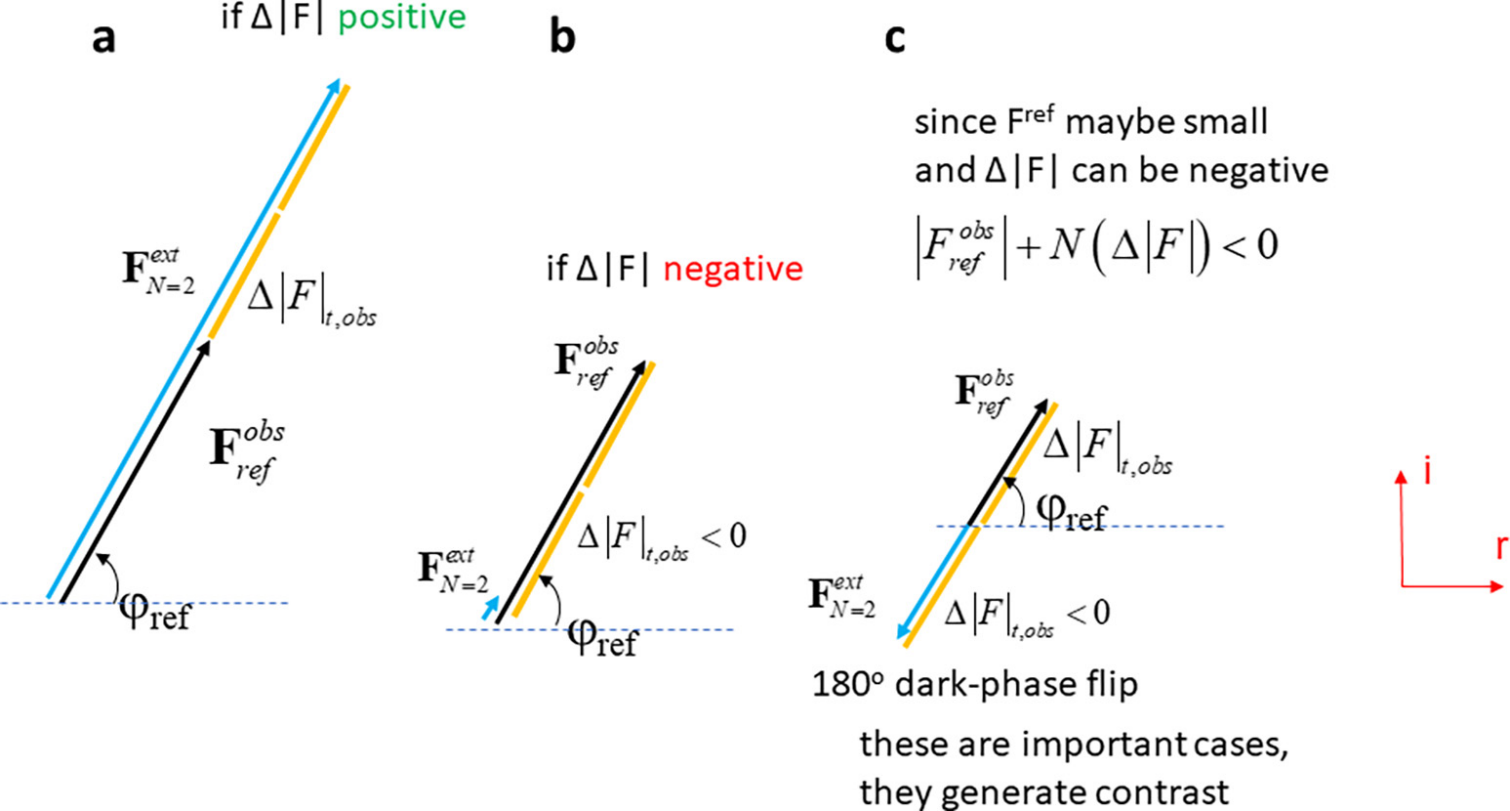
Extrapolated structure factors determination in the complex plane. N = 2 is applied for this example. (a) Δ|F| (orange) is positive. A structure factor with large magnitude and the dark phase emerges. (b) Δ|F| (orange) is negative. A structure factor with small amplitude and the dark phase emerges. (c) Δ|F| (orange) is negative, and **F**_ref_ is small. A structure factor **F**^ext^ with a moderate amplitude and a phase that opposes the dark state phase emerges.

This equation is unusual unless one accepts that the Fourier synthesis [Eq. (23)] can be executed with negative amplitudes. Again, from crystallographic first principles (complex number algebra), any negative amplitude can be replaced with a positive amplitude when the associated phase is flipped by 180°. Figure 6 explains the reason using a representation in the complex plane. The extrapolated structure factor amplitudes are calculated by aligning the difference structure factor amplitudes with the dark state structure factor. If the $\Delta {\left|F\right|}_{t}$ are positive, an extrapolated structure factor with a magnitude larger than the reference amplitude and with the reference phase emerges. The $\Delta {\left|F\right|}_{t}$ can also be negative. Then, an extrapolated structure factor with a smaller magnitude [Fig. 6(b)] can be obtained. However, and this is inevitable, some of the extrapolated structure factor amplitudes calculated from Eq. (22) will become negative. This situation is depicted in Fig. 6(c). The resulting extrapolated structure factor points in the opposite direction compared to the reference structure factor. This means that this extrapolated structure factor is an ordinary structure factor (with a positive amplitude), but its phase is φ_ref_ + 180°. Of course, it is this positive amplitude that must be submitted to a reciprocal space refinement program, and all extrapolated structure factors (with either the reference phase or the reference phase flipped by 180°) must be used to calculate an extrapolated map.

**FIG. 7.**
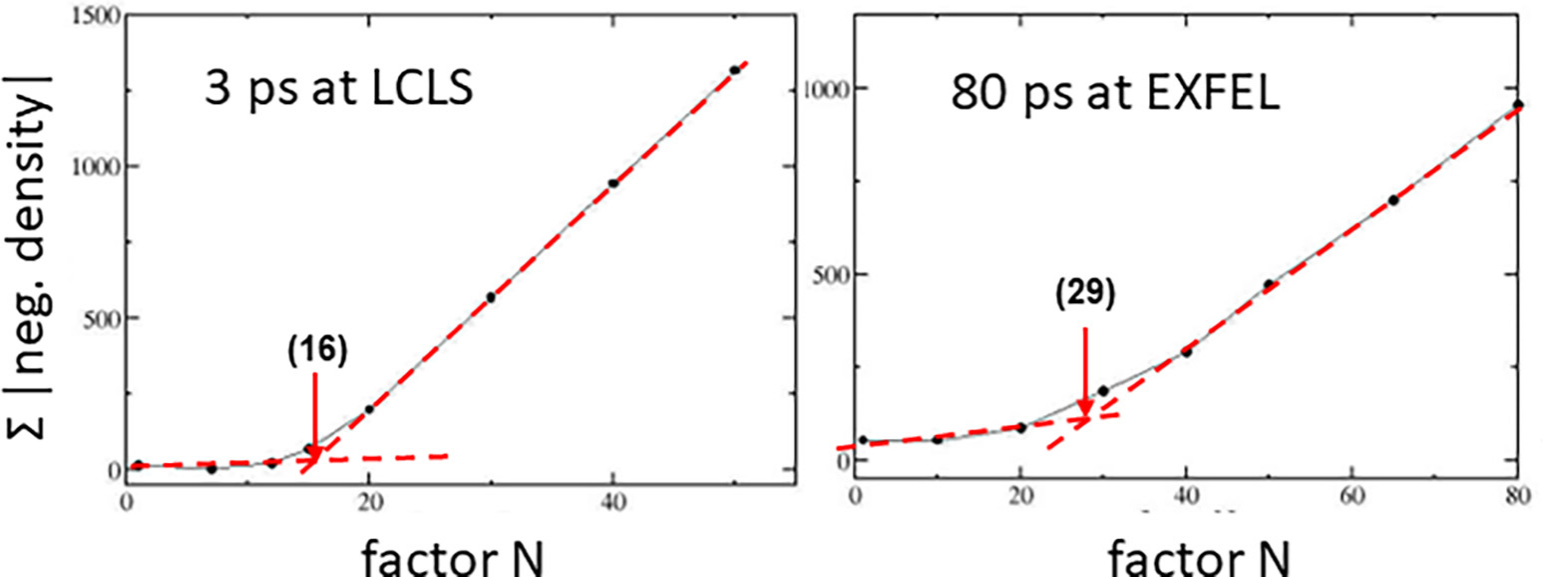
Determination of the characteristic N_C_ to determine extrapolated structure factors and maps. The negative density in a region of the extrapolated maps is integrated and plotted as a function of N. (a) N_C_ = 16 (∼12% occupancy) from an experiment on PYP at the LCLS (Pande *et al.*, 2016). (b) N_c_ = 29 after reaction initiation by femtosecond laser pulses (∼7% occupancy) at the European XFEL (EXFEL). The transition is marked with an arrow. The red lines are guides to the eye.

By omitting the “negative” structure factors from the Fourier summation [Eq. (23)] as suggested recently (De Zitter *et al.*, 2022), an extrapolated map (ρ_nn_, nn for *n*o-*n*egatives) is obtained that on the first glimpse appears quite similar to the correct DED map ρ_t_. Structural refinement against ρ_nn_, though, becomes more difficult. As an example, the ρ_nn_ map has been used to real-space refine the structure of the pCA chromophore [Fig. 5(b)] in photoactive yellow protein (PYP) with the goal to reproduce the torsional angle Φ_T_ determined previously (Pande *et al.*, 2016) [Fig. 5(b), red line]. To determine a structure that follows the electron density is more difficult and the result was quite different (Φ_T_ ∼140°) from that where all structure factor amplitudes are maintained (Φ_T_ ∼35°) (Pande *et al.*, 2016). For the calculation of ρ_ext_ in this example, the average extrapolated structure factor amplitude pointing in the direction of **F**_ref_ was 255 × f (f is the Thomson scattering length of an electron), whereas the average amplitude pointing into opposite direction to **F**_ref_ was 67 × f, a magnitude that cannot be neglected. It seems to be so that when the “negative” amplitudes are dismissed, it is not clear (a) whether an accurate characteristic N (N_C_) can be determined (see next paragraph for methods to determine N_C_), (b) whether the obtained extrapolated map is correct, and (c) whether a reciprocal space refinement against the incomplete |F^ext^| data will provide accurate structural displacements or structural relaxations. In any case, there is no physical reason to dismiss any extrapolated amplitudes. When an N_C_ is determined for the PYP data (Pande *et al.*, 2016; Pandey *et al.*, 2020), the fraction of structure factors pointing in the opposite direction to those of the reference is about 25% of all structure factors in the dataset whether N_C_ = 16 [Fig. 7(a)] or N_C_ = 29 [Fig. 7(b)] is employed. This large fraction might also have a deeper meaning that merits investigation.

## SEMI-AUTOMATIC DETERMINATION OF A CHARACTERISTIC FACTOR N_C_ TO CALCULATE AN EXTRAPOLATED MAP

A characteristic factor N_C_ must be determined to calculate an extrapolated map unless a faithful estimate of the occupancy is available from other sources. As shown by Terwilliger and Berendsen (Terwilliger and Berendzen, 1996), a well determined calculated electron density ρ_calc_ can replace the observed reference electron density in Eq. (21). Then, the reciprocal space equivalent would look like

$$\left|F^{\text{ext}}\right|=\left|F_{\text{ref}}^{\text{calc}}\right|+N_{C}\left(\Delta {\left|F\right|}_{t}\right),$$ (24)

with $\left|F_{\text{ref}}^{\text{calc}}\right|$ the structure factor amplitudes from a well refined dark state model. Once the characteristic N_C_ is determined, the occupancy of the intermediate can be calculated (see above) as

$$\text{occupancy}=\frac{2}{N_{C}}.$$ (25)

In order to obtain the N_C_ several methods can be applied. Here, three are listed: method 1 tries to isolate a well separatable volume with positive or negative difference electron density and integrates the difference electron density in this volume (Šrajer *et al.*, 2001). This integration provides an electron count, e.g., 1.5 e^−^. Due to the difference Fourier approximation, this value is on ½ the absolute scale. The true electron count is 3 e^−^. If a ligand has 14 electrons (e.g., carbon monoxide), this corresponds to about 21% occupancy. The factor N_c_ in this case would be about 10 [Eq. (25)].

**FIG. 8.**
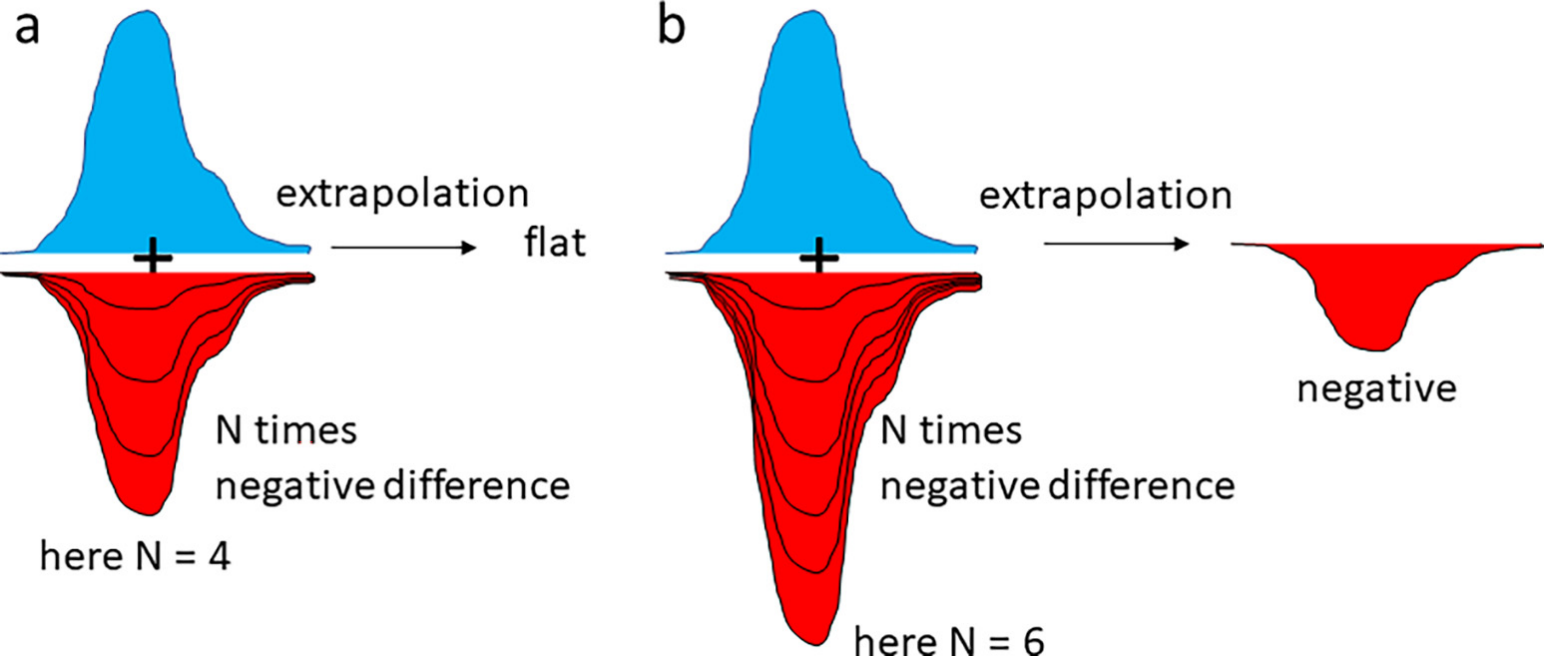
A region with strongly negative DED features. (a) The extrapolated map calculated with N = 4 is flat in this region. (b) When N = 6 is used, negative extrapolated electron density appears. This shows that N = 6 is too large. The characteristic N_C_ is closer to 4.

Method 2 uses the extrapolated maps themselves (Tripathi *et al.*, 2012; Schmidt, 2019; and Pandey *et al.*, 2020). A set of extrapolated structure factor amplitudes can be calculated with increasing factor N. Then, regions with strong negative density in the DED map become negative also in the extrapolated map [Fig. 8(b)]. These regions of interest (ROI) can be used to determine an accurate N_C_. Figure 7 shows real world examples from TR-SFX experiments at the LCLS and the European XFEL (EXFEL). With increasing N, the negative densities found in the ROIs of the extrapolated maps increase. The results are plotted as a function of N and the N_C_ determined at the intersection of the two red lines in Fig. 7(a) or Fig. 7(b).

Method 3 correlates calculated difference electron density features to the observed difference density features. With increasing N, structural models M_N_ can be refined against the resulting extrapolated structure factor amplitudes. From the resulting model, structure factors can be determined that can be used with the structure factors from the reference model to calculate a M_N_–M_ref_ difference map. Difference features are compared and correlated with the observed difference features (Claesson *et al.*, 2020). The correct N is found when the correlation between the two sets of difference features (observed and calculated) is optimum. Such an analysis can be performed in a user-friendly way using the program Xtrapol8 (De Zitter *et al.*, 2022). The author acknowledges a poster presentation by De Zitter *et al.* at the 2023 PSB symposium in Grenoble, Fr.

Once a characteristic N_c_ is determined, an extrapolated map can be calculated that allows for the determination and a real space refinement of a structural model, e.g., in Coot (Emsley *et al.*, 2010). An example of an extrapolated map from a recent experiment on photoactive yellow protein (PYP) is shown in Fig. 5(b) together with the corresponding difference map [Fig. 5(a)]. The ROI where the negative electron densities in the extrapolated maps are integrated is denoted by the dashed circle in Fig. 5(b). N_C_ = 16 has been determined from Fig. 7(a). If N_C_ would have been determined very different from 16 (for example N_C_ = 8), the torsional angle Φ_T_ would likely be substantially different. Since the torsional angle is a functional reaction coordinate for the PYP chromophore *trans* to *cis* isomerization, it is of paramount importance to determine this angle correctly. It has been suggested to improve the extrapolated map by density modification, such as solvent flattening and histogram matching, for example, using the program “dm'”(Cowtan, 1994). An improved structural model can then be determined from such a map and refined against the |F^ext^| (see also below).

## LOVE-HATE RELATIONSHIP WITH LARGE N_C_

Large extrapolation factors are a nuisance. Reciprocal space refinement against the extrapolated structure factor amplitudes (all of them, see discussion above) usually results in inacceptable R_cryst_ values of > 40%. Here, a way to remedy this is described.

The largest contribution to the error in the extrapolated structure factors is not the noise in the (measured) difference structure factor amplitudes, but the phase error introduced when adding the difference amplitudes to the dark state structure factors. The true phase φ_|ΔF|,true_ is simply not known (see also Figs. 2 and 3). Regardless whether or not density modification is applied, a structural model becomes available from the real-space fit to the (admittedly) noisy extrapolated map. This model and the model of the reference state can be used to calculate difference structure factors with amplitude |ΔF|^calc^ and estimated true phases φ_|ΔF|,#_. When the calculated phases φ_|ΔF|,#_ are combined with the Δ|F|^obs^, phased extrapolated structure factors are determined,

$$F^{\text{ext}}=\left|F_{\text{ref}}^{\text{calc}}\right|e^{i\varphi_{\text{ref}}}+N{\left|\Delta F_{t}\right|}^{\text{obs}}e^{i\varphi_{\Delta F,\#}}.$$ (26)

As a detail: by the application of the estimated phase of the difference structure factor φ_|ΔF|,#_, Δ|F|^obs^ becomes an ordinary amplitude |ΔF|^obs^ (note the position of the vertical straight lines to denote absolute values of the difference structure factor which is used [compare Eqs. (24) and (26)]. The calculation is particularly easy, if the Δ|F|^obs^ are stored as positive values for book-keeping [Eq. (17)]. From these, a phased extrapolated electron density map is calculated. Refinement against the phased extrapolated structure factor amplitudes immediately results in acceptable R_cryst_ values.

**FIG. 9.**
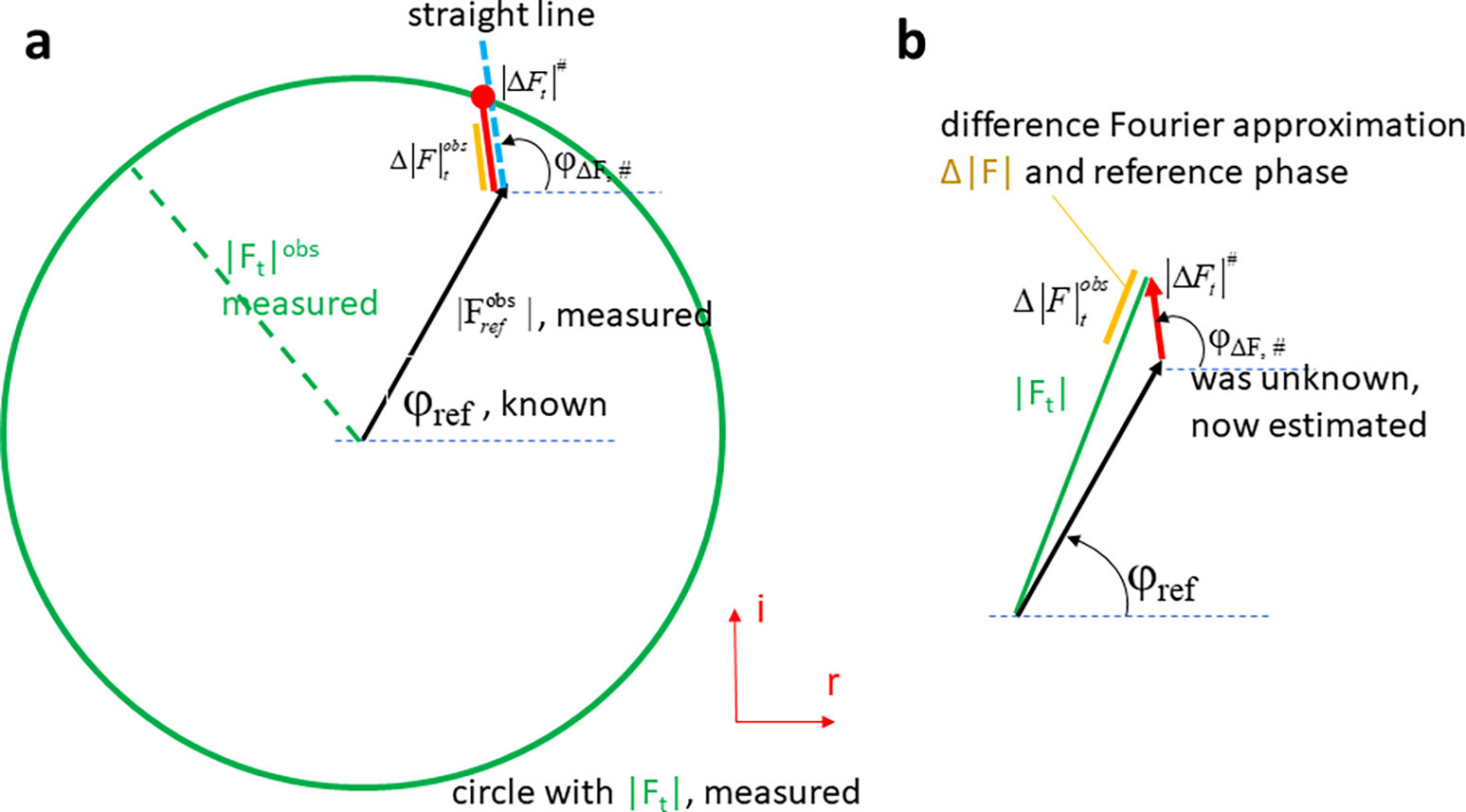
Correcting the difference Fourier approximation. Argand diagram to estimate the true length (red) of a difference structure factor amplitude that has been previously determined by projection (orange). (a) The true phase, φ_|ΔF|,#_, of the difference structure factor can be estimated once structural models of an intermediate and the reference state are available. The blue line points along the φ_|ΔF|,#_. By circling about the origin with the measured time-dependent structure factor amplitude (green), the triangle closes at the red dot. The closure is again shown in (b). An estimate of the true difference structure factor (in red) with amplitude and phase in relationship to the projected difference structure factor amplitude (orange) is depicted.

A more sophisticated method to recover the magnitude of the true difference structure factor amplitude that was previously only estimated by projection [Eq. (10)] is shown in Fig. 9. Here, the situation is depicted by an Argand diagram that makes use of the estimated true phase φ_|ΔF|,#_. The orange difference is the weighted difference structure factor, and the red difference is a corrected difference structure factor ${\left|\Delta F_{t}\right|}^{\#}$ that closes the triangle between the measured time-dependent amplitude and the sum of the dark structure factor and the difference structure factor. Extrapolation [Eq. (26)] can then be pursued with the corrected ${\left|\Delta F_{t}\right|}^{\#}$ and the phase ϕ_|ΔF|,#_. Since the difference Fourier approximation is not applied, the reason for the factor 2 in [Eqs. (21) and (22)] vanishes. N becomes much smaller, and the extrapolated map appears much improved.

The method shown in Fig. 9 needs closer examination. In particular, the probability of the true difference structure factor given the noise and other systematic errors in the data and the structural models must be evaluated perhaps in a similar way as it has been done previously for partial structural models with errors (Read, 1986, 1997). In addition, the phase bias introduced by a model refined against the extrapolated map needs to be estimated. The best way to do this is by engaging an appropriate simulation using realistic structure factors with noise followed by a statistical analysis as performed previously (Read, 1986; Schmidt *et al.*, 2003).

It can only be hoped that this tutorial will help to promote the widespread usage of TRX methods. Appropriate software solutions will be user-friendly with push-button interface and more functional in the future so that everyone with general crystallography knowledge can learn and practice structure determination from time-resolved DED maps.

## Data Availability

No data were generated for this manuscript.
